# Improved Physicochemical Stability and High Ion Transportation of Poly(Arylene Ether Sulfone) Blocks Containing a Fluorinated Hydrophobic Part for Anion Exchange Membrane Applications

**DOI:** 10.3390/polym10121400

**Published:** 2018-12-17

**Authors:** Ji Young Chu, Kyu Ha Lee, Ae Rhan Kim, Dong Jin Yoo

**Affiliations:** 1 Department of Energy Storage/Conversion Engineering of Graduate School, Hydrogen and Fuel Cell Research Center, Chonbuk National University, Jeonju 54896, Korea; ebbuneg@hanmail.net (J.Y.C.); carumiss@naver.com (K.H.L.); 2 R&D Center for CANUTECH, Business Incubation Center and Department of Bioenvironmental Chemistry, Chonbuk National University, Jeonju 54896, Korea; 3 Department of Life Science, Chonbuk National University, Jeonju 54896, Korea

**Keywords:** quaternized PAES membrane, hydroxide conductivity, ion cluster, alkaline stability, alkaline fuel cells

## Abstract

A series of anion exchange membranes composed of partially fluorinated poly(arylene ether sulfone)s (PAESs) multiblock copolymers bearing quaternary ammonium groups were synthesized with controlled lengths of the hydrophilic precursor and hydrophobic oligomer via direct polycondensation. The chloromethylation and quaternization proceeded well by optimizing the reaction conditions to improve hydroxide conductivity and physical stability, and the fabricated membranes were very flexible and transparent. Atomic force microscope images of quaternized PAES (QN-PAES) membranes showed excellent hydrophilic/hydrophobic phase separation and distinct ion transition channels. An extended architecture of phase separation was observed by increasing the hydrophilic oligomer length, which resulted in significant improvements in the water uptake, ion exchange capacity, and hydroxide conductivity. Furthermore, the open circuit voltage (OCV) of QN-PAES X10Y23 and X10Y13 was found to be above 0.9 V, and the maximum power density of QN-PAES X10Y13 was 131.7 mW cm^−2^ at 60 °C under 100% RH.

## 1. Introduction

Fuel cells are energy conversion devices that convert chemical energy into electrical energy while being supplied with fuel and oxidant. They generate electricity with high conversion efficiency, high energy density, and low pollution emission levels. Proton exchange membrane fuel cells (PEMFCs), which is one of the types of fuel cells, have the advantages of satisfactory cell performance and a wide range of applications [1,2,3]. However, commercialization of PEMFCs has been impeded by (i) the high costs of materials (i.e., perfluorinated membranes and precious metal catalysts) and (ii) poor long-term stability during cell operation above 90 °C [4,5,6,7]. On the other hand, alkaline fuel cells (AFCs) have a faster electrochemical reaction mechanism in alkaline media as well as a low over potential for fuel oxidation; AFCs also utilize non-precious metals (Ni, Co) as electrode catalysts; thus, AFC is being developed as a preferable alternative to PEMFC [8,9,10,11,12].

Initially, liquid electrolytes (KOH and NaOH) were used in AFC, but the development of a solid electrolyte membrane has been actively pursued because carbonate salts (formed from the reaction of CO_2_ in the air with KOH) block the pores of the catalyst electrode. These salts affect the durability and cell performance, resulting in additional problems such as wetting, increased corrosion, and difficulty in handling pressure differences. Therefore, over the past decades, studies of anion exchange membranes (AEMs) as a barrier for fuels/oxidants, and hydroxyl ion transfer carriers have been ongoing. However, there are problems associated with low hydroxide conductivity and inferior chemical stability, which prevent the commercialization of AEMs in AFC. Hydroxide conductivity in AEMs is known to be lower than that of proton exchange membranes (PEMs) due to the low mobility of OH^−^ ions, and the performance of AEM tends to degrade due to severe exposure in OH^−^ at high pH conditions. Therefore, a high ion exchange capacity (IEC) value is essential to obtaining high ionic conductivity and fuel cell performance for applications in AEM. At present, a variety of AEMs having main polymer backbone materials such as poly(olefin)s, poly(arylene ether)s, poly(arylene ether sulfone)s, poly(ether imide)s, poly(arylene ether ketone)s, and poly(phenylene oxide)s are being designed [13,14,15,16,17,18,19,20]. As previously mentioned, the most important part of the AFC components is to synthesize AEMs having high hydroxide conductivity and improved mechanical strength. In recent research, micro phase-separated block copolymers with controlled hydrophilic and hydrophobic oligomer lengths exhibited improved ionic conductivity and thermal stability. Pan et al. [21] studied the ion aggregating ability of different structures and demonstrated that the hydroxide conductivity could be enhanced by the formation of larger ionic clusters and interconnected broad ionic channels. Functional groups as well as morphologies are key factors to improving the electrochemical performance in AEMs. Different types of cations including guanidinium [22,23,24], imidazolium [25,26,27,28,29], phosphonium [30], pyrrolidinium [31,32], piperidinium [33], and metal cations [34] have been extensively studied. However, among the many functional groups, alkyl amines (having low cost and good performance) are widely used in many cases because other functional groups are costly. Based on the above methods, an AEM exhibiting high IEC with improved ionic conductivity may suffer from excessive dimensional changes and decreased mechanical strength. Therefore, it is essential to consider the polymer backbone design of AEM to achieve proper mechanical stability and optimized ionic conductivity. Dhara et al. [17] reported block copolymers with perfluorinated substituents. Strong C–F bonds have good thermal stability, improved chemical resistance, lower surface energy, and improved solubility associated with polymer processing, and can lead to easy synthesis due to their very high reactivity. In addition, fluorine units with strong hydrophobic properties are highly nonpolar, leading to high ionic conductivity by pushing water to the hydrophilic regions and ensuring the ion pathways. It has also been reported that the bulky –CF_3_ structure can control the permeation of fuel by increasing the free volume in the polymer and reinforcing the flexibility of the membrane due to having a sp^3^ hybrid structure.

In line with these facts, partially fluorinated PAES block copolymers were synthesized with different lengths of hydrophilic precursor and hydrophobic oligomer. The block copolymers containing a fluorinated hydrophobic portion were designed for the purpose of improved ionic conductivity, physicochemical stability, and flexibility of the membrane. The morphologies of the PAES were controlled to produce a polymer structure that has excessive ion clusters. The successful introduction of the quaternary ammonium groups (chloromethylation) into a specific position of the fluorenyl group was carried out via the Friedel-Crafts reaction. The chloromethylation proceeded effectively by optimizing the reaction conditions. The chemical structure of the synthesized PAES, CM-PAES, and QN-PAES was confirmed through ^1^H NMR, FT-IR, and GPC. Furthermore, characterizations of the prepared AEMs including thermal stability, mechanical properties, dimensional stabilities, hydroxide conductivity, and alkaline stabilities were studied in detail.

## 2. Experimental

### 2.1. Materials

4,4′-(9-Fluorenylidene)diphenol (BPFL), bis(4-fluorophenyl)sulfone (FPS), chloromethyl methyl ether (CMME), anhydrous *N*,*N*-dimethylacetamide (DMAc), anhydrous toluene, and dimethyl sulfoxide-d_6_ (DMSO-d_6_) were purchased from Sigma-Aldrich (Saint Louis, MI, USA). 4,4′-(Hexafluoroisopropylidene)diphenol (6F-BPA), decafluorobiphenyl (DFBP), and 45 wt % trimethylamine aqueous solution were purchased from Alfa Aesar (Haverhill, MA, USA). The zinc chloride (ZnCl_2_), potassium carbonate (K_2_CO_3_), 1,1,2,2-tetrachloroethane (TCE), tetrahydrofuran (THF), methanol, and acetone were of reagent grade.

### 2.2. Synthesis of QN-PAES Membranes

#### 2.2.1. Synthesis of Poly(arylene sulfone) (PAS) Hydrophilic Precursors

The PAS hydrophilic precursors were synthesized via a polycondensation reaction with BPFL and FPS, and the PAS-X10 and -X19 (where X represents repeat units of PAS hydrophilic precursor) were prepared by controlling the reaction time. Typically, the PAS-X10 was obtained as follows: BPFL (2.85 mmol), FPS (2.59 mmol), K_2_CO_3_ (6.27 mmol), and anhydrous DMAc/toluene (20 mL/20 mL) were put together into a 100 mL round-bottomed flask equipped with a Dean-Stark trap and nitrogen gas inlet. After the mixture was homogeneously mixed at 110 °C for 1 h, the mixture temperature was increased to 170 °C, and kept for 18 h. Afterwards, the mixture was cooled to room temperature (RT), and viscous solution was slowly poured into a deionized water (DI water)/acetone/methanol (*v*/*v*/*v*, 1/1/7) solvent. The precipitated powder was repeatedly filtered and dried in an oven at 70 °C for more than 24 h. ^1^H NMR (600 MHz, DMSO-*d_6_*): 6.75–7.40 (18H), 7.69–7.95 (6H).

#### 2.2.2. Synthesis of Fluorinated Poly(arylene ether) (PAE) Hydrophobic Oligomers

The PAE oligomers (-Y13 and -Y23, where Y represents repeat units of PAE oligomer) were prepared by a polymerization reaction. Typically, PAE-Y13 was obtained as follows. DFBP (2.99 mmol), 6F-BPA (2.72 mmol), K_2_CO_3_ (5.98 mmol), and 30 mL of anhydrous DMAc were mixed in a round-bottomed flask equipped with a condenser and nitrogen gas inlet. The mixture was stirred at RT for 1 h, then the mixture temperature was raised to 120 °C and held for 18 h. Afterwards, the mixture was cooled at RT and slowly poured in to a methanol/DI water (*v*/*v*, 4/1) solvent to produce a solid powder. The precipitate was collected by filtration and dried in an oven at 70 °C for 24 h. ^1^H NMR (600 MHz, DMSO-*d*_6_): 7.30–7.42 (8H).

#### 2.2.3. Polymerization Reaction with PAS Hydrophilic Precursor and PAE Hydrophobic Oligomer

The synthetic procedure for the PAES X10Y13 block copolymer was as follows: The round-bottomed flask equipped with a condenser was charged with PAS-X10 hydrophilic precursor (30 mmol), PAE-Y13 (30 mmol), K_2_CO_3_ (60 mmol), and anhydrous DMAc (20 mL) under nitrogen gas flow. The mixture was heated to 120 °C and held at that temperature for 24 h. After the mixture was cooled at RT, it was slowly poured into DI water/methanol (*v*/*v*, 2/3) solvents to recrystallize the viscous solution. The precipitate was collected by filtration and dried in a vacuum oven at 70 ℃ for 24 h. ^1^H NMR (600 MHz, DMSO-*d_6_*): 6.60–7.42 (26H), 7.69–7.95 (6H).

#### 2.2.4. Chloromethylation of PAES Block Copolymers

**Scheme 1:**
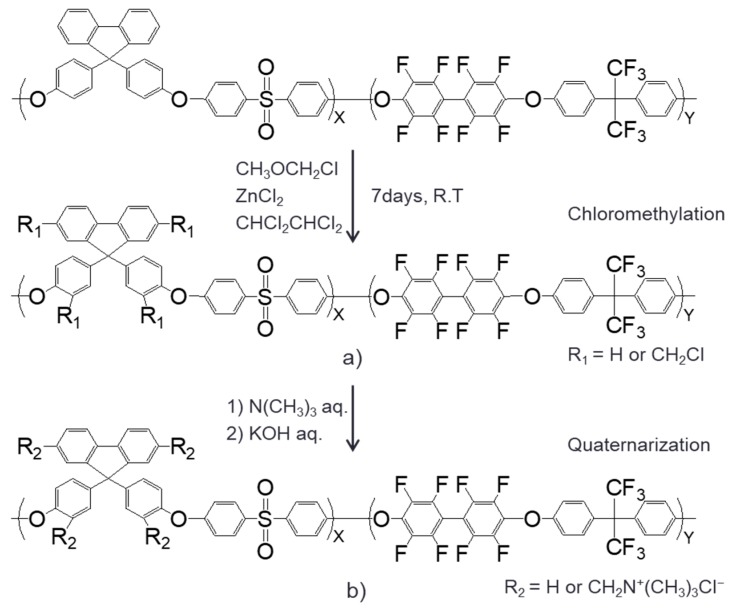
Synthetic steps to prepare (**a**) CM-PAES X10Y13 and (**b**) QN-PAES X10Y13.

The CM-PAES X10Y13 block copolymer was obtained by a typical procedure as follows (Scheme 1a): The round-bottomed flask equipped with a dropping funnel was charged with 1.00 g (1.77 mmol) of PAES X10Y13 and TCE (40 mL) under nitrogen gas flow. After these were completely dissolved, ZnCl_2_ (0.24 g, 1.77 mmol) in THF (5 mL) and CMME (6 mL, 79 mmol) (as the chloromethylation agent) were slowly added to prevent undesirable reactions. The mixture temperature was raised to 50 °C and was held for 7 days. The viscous solution was poured into 800 mL methanol, and then the solid precipitate was collected by filtration. Finally, the obtained powder was dried in an oven at 55 °C for 24 h.

#### 2.2.5. Fabrication of Membranes, Quaternization, and Anionization

**Figure 1:**
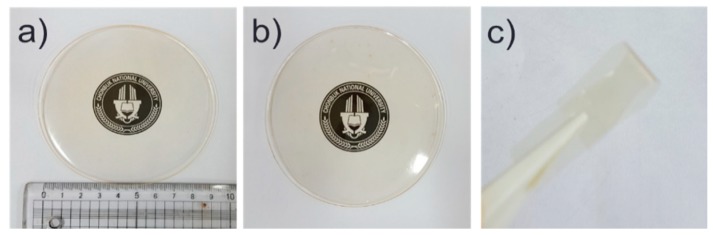
Photographs of membranes; (**a**) CM-PAES X10Y13, (**b**) QN-PAES X10Y13, and (**c**) bending QN-PAES X10Y13.

The QN-PAES X10Y13 membrane was obtained by following a typical procedure (Scheme 1b): Briefly, CM-PAES X10Y13 (0.5 g) was dissolved in TCE (20 mL). After completely dissolving, the mixture was directly cast on a petri dish and dried at 70 °C. The fabricated CM-PAES membranes were peeled off using DI water and were immersed in a 45 wt% (*w*/*w*) trimethylamine aqueous solution at 30 °C for 48 h to convert Cl groups to quaternary ammonium groups. The QN-PAES X10Y13 membrane was soaked in a 1 M KOH solution for 24 h and then kept in DI water to prevent CO_2_ contamination. As shown in Figure 1, the fabricated QN-PAES X10Y13 membranes with thicknesses of 30 ± 5 µm were transparent and flexible without any significant visible defects.

### 2.3. Chraterizations

The ^1^H NMR spectra were analyzed by JNM-ECA 600 instrument (JEOL, Akishima, Tokyo, Japan). The IR spectra were obtained by Frontier MIR/NIR spectrometer (PerkinElmer, Waltham, MA, USA). The molecular weights (*M*_n_, *M*_w_, and *M*_z_) and polydispersity indices (PDI, *M*_w_/*M*_n_) were measured by HLC-8320 (Tosoh Corporation, Minato, Tokyo, Japan). The thermogravimetric analysis (TGA) was conducted by Q 50 (TA Instruments, New Castle, DE, USA). The tapping mode-atomic force microscopy images were conducted by a Veeco multimode atomic force microscope (AFM, Veeco Corporation, Plainview, NY, USA). The mechanical behaviors was measured by LR5K-plus (Ametck Lioyd Instruments, Indianapolis, Indiana, UK). The hydroxide conductivity was measured by the conductivity test bench (Scitech Korea, Gangbuk, Seoul, Korea). The average ion domain size was recorded by using SAXS (EMPYREAN, Malvern Panalytical Ltd, Malvern, Grovewood, UK) [30]. The fuel cell performance was measured using single cell test station (Scitech, Gangbuk, Seoul, Korea).

The water uptake and swelling ratio was evaluated using dried and wet membrane. The water uptake value of membranes was reported using the following equation:

Water uptake (%) = (W_wet_ − W_dry_)/W_dry_ × 100%, (1)

The dimensional ratio was calculated using this equation:

Swelling ratio (%) = (S_wet_ − S_dry_)/S_dry_ × 100%, (2)

The ion exchange capacity (IEC) of QN-PAES membranes was measured by the back-titration method [21,35,36]. Samples (10 mm × 10 mm) of the QN-PAES (OH^−^ form) membrane were soaked in 50 mL of 1 M HCl solution at RT for 48 h to convert the OH^−^ form to the Cl^−^ form. Then, the consumed HCl was titrated using 0.1 N NaOH with phenolphthalein as an indicator. The IEC value was calculated using the following equation:

IEC (mequiv g^−1^) = (V,B_HCl_ − V,A_HCl_)/W_dry_, (3)

where V,B_HCl_ and V,A_HCl_ are the mequiv (mmol) of HCl before and after equilibrium, respectively, and W_dry_ is the weight of the dry membrane.

The chemical stability was evaluated by soaking them in a 2 M NaOH solution at 70 °C for 600 h. The samples were taken out of the alkaline solution and washed repeatedly with DI water for the alkaline stability test. Then, the hydroxide conductivity and IR spectra of the samples were monitored at intervals of 120 h during the alkaline stability test.

## 3. Results and Discussion

### 3.1. Synthesis and Characterization of PAS, PAE, and PAES Polymer

**Figure 2:**
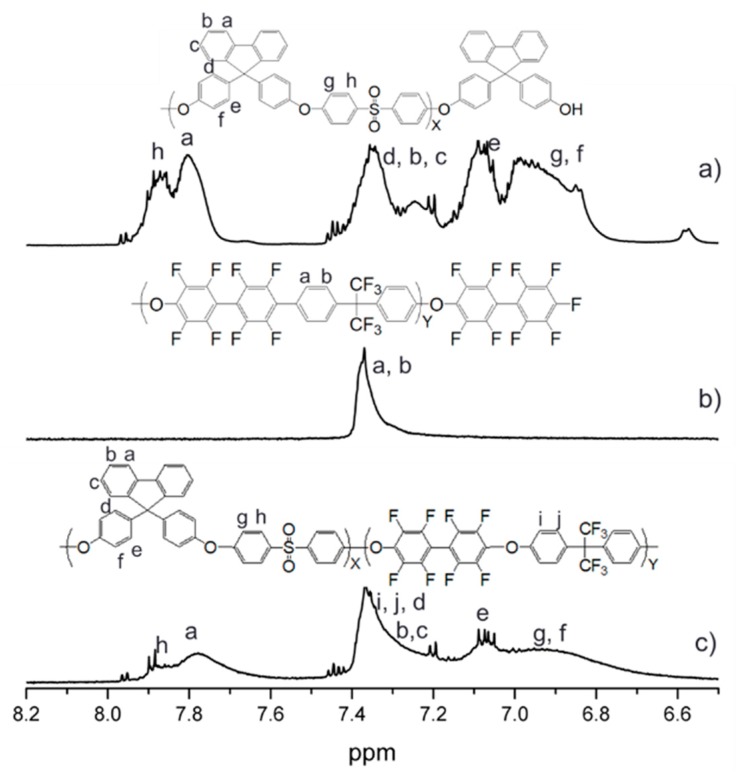
^1^H NMR of (**a**) PAS-X10, (**b**) PAE-Y13, and (**c**) PAES X10Y13.

PAES X*n*Y*m* block copolymers (X10Y23, X10Y13, X19Y23, and X19Y13) were synthesized via a direct polymerization reaction. The chemical structures of the synthesized polymers were investigated by GPC (Table 1) and ^1^H NMR (Figure 2 and Figure S1). The polymer lengths of the PAS hydrophilic precursors were determined by GPC data and an integral ratio between the proton peaks of the polymer backbone and the terminal end groups were determined from ^1^H NMR spectra (Figure S1). The calculated repeating units of the PAS hydrophilic precursor obtained from GPC data well matched the calculated repeating units of the PAS hydrophilic precursor obtained from ^1^H NMR data. The GPC data of hydrophilic precursors and hydrophobic oligomers exhibited a low PDI value in the range from 1.6 to 2.3, suggesting that no unfavorable polymer side reactions occurred during the polymerization reaction. The repeat unit of the PAS hydrophilic precursor and PAE hydrophobic oligomers was calculated from GPC data [37] and is shown in Table 1.

The block copolymerization reaction was conducted with varying lengths of PAS hydrophilic precursors and PAE hydrophobic oligomers to synthesize a series of PAES multiblock copolymers (X10Y23, X10Y13, X19Y23, and X19Y13). The prepared PAES block copolymers were readily soluble in a variety of organic solvents including DMSO, DMAc, *N,N*-dimethylformamide (DMF), TCE, and *N*-methyl-2-pyrrolidone (NMP). The chemical structure of PAES X10Y13 was investigated from ^1^H NMR spectra (Figure 2c). The proton peaks of PAES X10Y13 were assigned to reference proton peaks of PAS-X10 and PAE-Y13; the ^1^H NMR spectra of PAES X10Y13 were observed in the range of 6.65−8.00 ppm. The molecular weights (*M*_n_ and *M*_w_) of PAES block copolymers were measured by GPC, and the values are summarized in Table 2. The molecular weights of the synthesized PAES block copolymers (*M*_w_ > 87.5 kDa, *M*_n_ > 19.8 kDa) were much higher than the hydrophilic precursors and hydrophobic oligomers, indicating the successful synthesis of the multiblock copolymers.

### 3.2. Synthesis and Characterization of CM-PAES and QN-PAES

**Figure 3:**
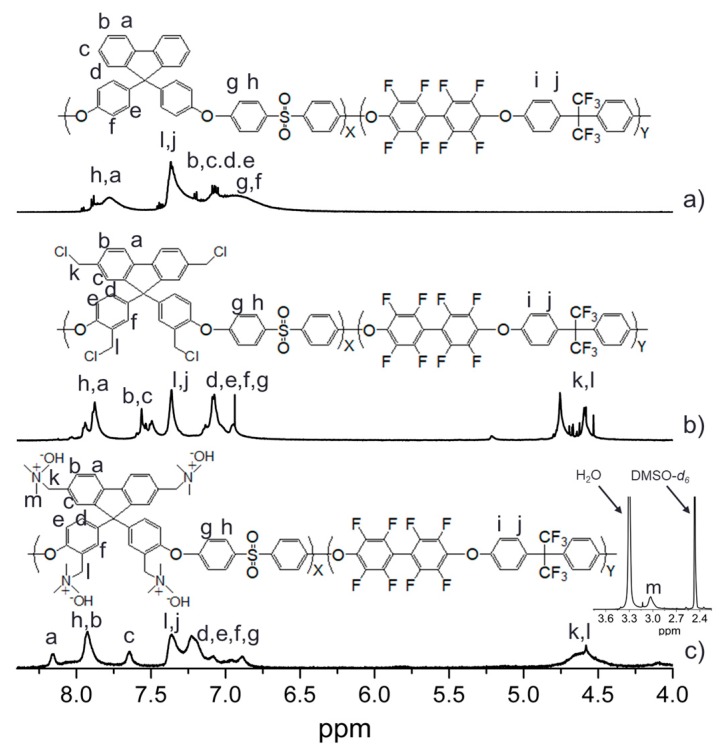
^1^H NMR of (**a**) PAES X10Y13, (**b**) CM-PAES X10Y13, and (**c**) QN-PAES X10Y13.

The CM-PAES was synthesized using CMME as a reactant and ZnCl_2_ as a Lewis catalyst in a TCE solution via Friedel-Crafts alkylation. The reaction conditions had to be carefully controlled since chloromethylation is often caused by undesirable reactions such as crosslinking and gelation of the mixture. The optimized conditions were applied to the synthesis of CM-PAES according to a previously reported paper [14]. Structural analysis of the CM-PAES X10Y13 and QN-PAES X10Y13 was carried out from ^1^H NMR spectra. In the ^1^H NMR spectra of CM-PAES X10Y13 (Figure 3b), the proton peaks of the fluorenyl unit due to the introduction of chloromethyl groups partially shifted between 6.8 and 7.7 ppm, and the methylene proton peaks (denoted as H_k,l_ in Figure 3b) corresponding to the chloromethyl groups were newly observed between 4.4 ppm and 5.4 ppm; these were not observed in the ^1^H NMR spectra of PAES X10Y13. The integral ratio of the newly appeared proton peak between 4.4 and 5.4 ppm was coincident with that of a decreasing peak at 6.95 ppm. After chloromethylation, altered proton peaks were not found in the hydrophobic regions, and these results agree with a previously reported paper [38]. The degree of chloromethylation of CM-PAES block copolymers was calculated from the integral ratio of the unchanged proton peaks (denoted as H_a,h_ in Figure 3b) in the main chain and methylene proton peaks (denoted as H_k,l_ in Figure 3b) of chloromethyl groups. CM-PAES X10Y13 was dissolved in TCE and directly cast on a petri dish to prepare a membrane. The membrane of CM-PAES X10Y13 was converted to QN-PAES X10Y13 by immersion in a 45 wt % trimethylamine aqueous solution, and then the QN-PAES X10Y13 membrane was soaked in 1 M KOH solution for 24 h. The chemical structure of QN-PAES X10Y13 was characterized using ^1^H NMR spectra. The sharp methyl peak corresponding to chloromethyl groups on the fluorenyl groups, which appeared between 4.4 and 5.4 ppm in the ^1^H NMR spectra of CM-PAES, broadly appeared to one peak at similar positions in ^1^H NMR spectra of QN-PAES due to the strong electron-withdrawing effect of the quaternary ammonium groups. Also, proton peaks (denoted as H_m_ in Figure 3c) corresponding to substituted methyl groups of the ammonium groups are shown at 3.0 ppm.

**Figure 4:**
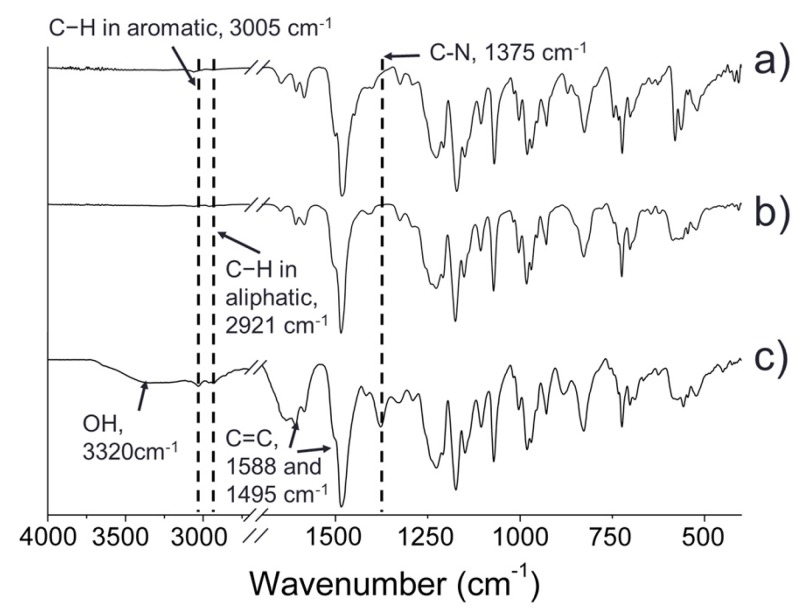
FT-IR spectra of (**a**) PAES X10Y13, (**b**) CM-PAES X10Y13, and (**c**) QN-PAES X10Y13.

In addition, the introduction of chloromethyl functional groups and quaternary ammonium groups on the polymer backbone was confirmed by FT-IR spectra. As shown in Figure 4, all the polymers exhibited a characteristic peak corresponding to the vibrations of aromatic C=C double bonds on the benzene rings at 1588 and 1495 cm^−1^, and the characteristic peak at 3005 and 2921 cm^−1^ in Figure 4b,c was attributed to stretching vibrations of aromatic and aliphatic C−H bonds in methyl or methylene groups, respectively. Additionally, after quaternization with trimethylamine, the characteristic stretching of the C−N bond of the ammonium functional groups was observed at 1375 cm^−1^ in Figure 4c. This indicates the presence of quaternary ammonium groups in the polymer main chain. Therefore, these results demonstrate that chloromethyl groups and quaternary ammonium groups were introduced successfully to the polymer backbone.

### 3.3. Thermal Stability

**Figure 5:**
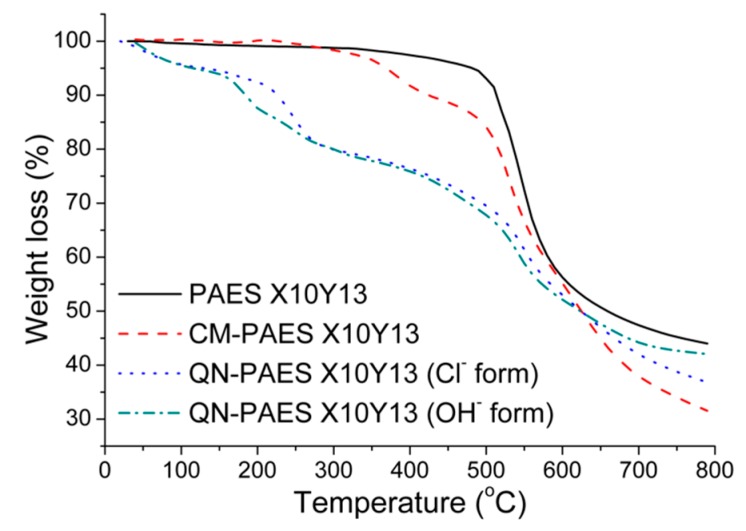
Thermal stability of polymers at heat rate of 10 °C min^−1^.

The thermal stability of the PAES X10Y13, CM-PAES X10Y13, and QN-PAES X10Y13 (Cl^−^ form and OH^−^ form) was evaluated by TGA analysis at a heating rate of 10 °C min^−1^ under a nitrogen atmosphere, and the results are shown in Figure 5. First, PAES X10Y13 was confirmed to be thermally stable through decomposition at over 500 ℃. The CM-PAES X10Y13 exhibited a first small weight loss corresponding to decomposition of chloromethyl functional groups at approximately 300 °C and a second weight loss corresponding to the degradation of main chains at 450 °C. The initial degradation of the QN-PAES X10Y13 (Cl^−^ form and OH^−^ form) block copolymer due to the residual organic solvent and tightly bound water molecules released in the membrane was induced below 120 °C, although the membranes were measured after completely drying. The second weight loss of the QN-PAES X10Y13 at 150–250 °C is related to the degradation of the quaternary ammonium groups. Finally, a third degradation around 400 °C, assigned to the decomposition of the polymer backbone TGA analysis of the synthesized polymers, show that the synthesized AEMs are well-suited for use as a commercial AEMs considering the alkaline fuel cell operating temperature is 80 °C.

### 3.4. Mechanical Behaviors

The mechanical behaviors of QN-PAES membranes were investigated by UTM (Table 3). QN-PAES membranes were prepared in a fully dried state to measure the mechanical properties. As shown in Table 3, the tensile behavior, Young’s modulus, and elongation at break of QN-PAES membranes were in the 60.5–77.6 MPa, 0.9–3.4 GPa, and 1.8−10.9% ranges, respectively. These results show that QN-PAES membranes are malleable for applications in membrane electrolyte assembly (MEA). Tensile stress and Young’s modulus decreased as the length of the hydrophilic precursors increased (and the length of hydrophobic oligomers decreased). In contrast, the elongation at break increased. This is because increasing the length of the hydrophilic precursors and decreasing the length of the hydrophobic oligomers enhanced water absorption, which altered the mechanical properties of the membrane. More importantly, the mechanical stability of QN-PAES membranes based on AEMs was higher than that of previously reported papers [33,38,39]. According to the above results, the fabricated QN-PAES membranes meet the mechanical properties required for applications in AEMFC.

### 3.5. Water Uptake, IEC, and Dimensional Properties

**Figure 6:**
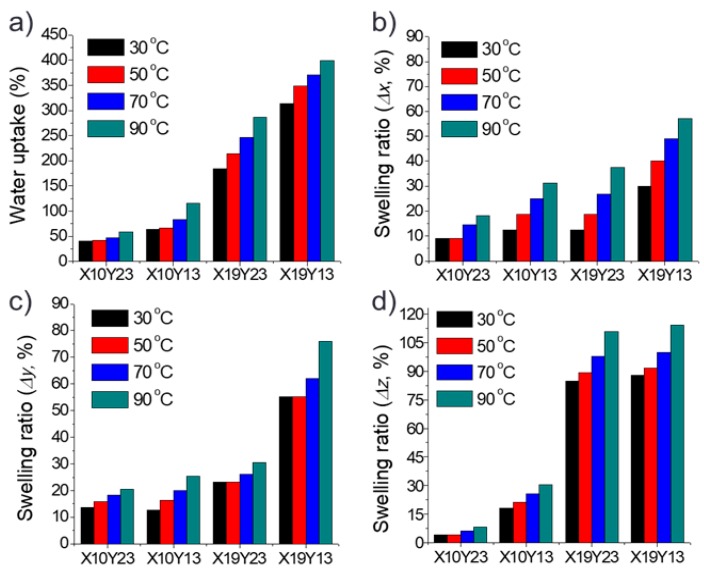
The (**a**) water uptake and dimensional stabilities (swelling ratio of (**b**) width (∆*x*), (**c**) length (∆*y*), and (**d**) thickness(∆*z*)) of QN-PAES membranes.

Water uptake is an important factor in determining fuel cell performance, due to its profound effect on the hydroxide conductivity and mechanical behaviors of AEMs. Water absorption is necessary to develop ionic clusters in the membrane. The membrane that absorbs a large number of water molecules allows fast OH^−^ transfer through the expanded ion channel, inducing a higher hydroxide conductivity. However, insufficient or excessive water absorption may cause lower hydroxide conduction or unstable mechanical properties of the membrane, respectively. Thus, the relationship between the water uptake and IEC values is crucial for developing AEMs. The fabricated QN-PAES membranes were pretreated in 1 M KOH over 30 h at RT to convert Cl^−^ to OH^−^ for the measurement of water uptake, IEC, and dimensional stability. The water uptake and dimensional stability of QN-PAES membranes were measured at various temperatures, and the values are summarized in Figure 6 and Table S1. As shown in Figure 6a and Table S1, the water uptake of QN-PAES membranes increased from 58.5% to 399.3%, with an increase in the IEC value at 90 °C. Dimensional stabilities (width (∆*x*), length (∆*y*), and thickness (∆*z*)) of QN-PAES membranes also increased from 18.2% to 57.0%, 20.5% to 75.9%, and 8.3% to 114.3% with an increase in the IEC value at 90 ℃, respectively. As expected, the QN-PAES X10Y23 membrane with a shorter hydrophilic precursor exhibited more suitable water uptake and dimensional stability than that of a QN-PAES X19Y23 membrane with the same hydrophobic length. The IEC values of QN-PAES membranes were in the range from 1.36 to 2.10 mequiv g^−1^ based on the controlled lengths of the hydrophilic precursor and hydrophobic oligomer (Table 4). These results indicate that water uptake and dimensional stability are associated with IEC values.

### 3.6. Morphology

**Figure 7:**
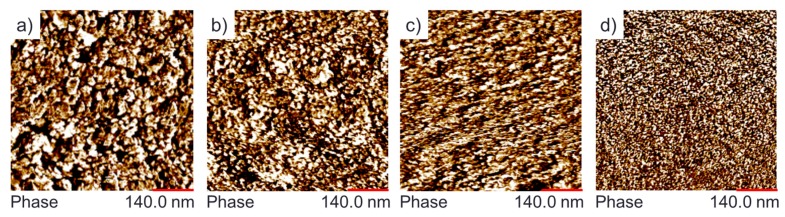
AFM phase images of (**a**) QN-PAES X10Y23, (**b**) QN-PAES X10Y13, (**c**) QN-PAES X19Y23, and (**d**) QN-PAES X19Y13.

**Figure 8:**
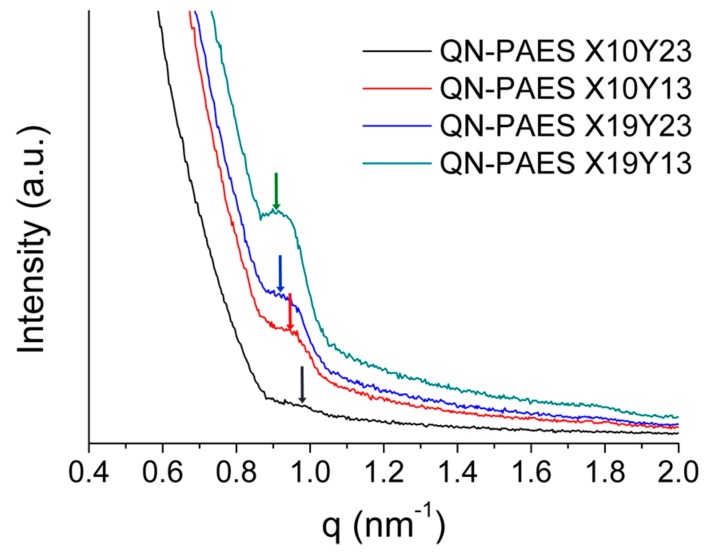
The SAXS patterns of QN-PAES membranes.

The microstructure of the QN-PAES membranes was investigated by AFM (Figure 7 and Figure S2). As shown in Figure 7, the morphologies of QN-PAES membranes showed distinct microphase separation of the hydrophilic/hydrophobic domain in which the bright regions correspond to the hydrophobic domains, while the dark regions are attributed to the hydrophilic domains (including the quaternary ammonium groups) [40,41,42,43,44,45]. The ion channels of QN-PAES membranes exhibit different conformations depending on the proportion of the hydrophilic/hydrophobic domains. Among the QN-PAES membranes, the morphology of QN-PAES X10Y23 exhibited a partially dense ion cluster and ion transfer channel, indicating poor connections between domains (Figure 7a). However, the morphology of QN-PAES X19Y13 showed connections between hydrophilic and hydrophobic domains unlike QN-PAES X10Y23. These results indicate that the ion channel becomes evenly distributed on a surface. Furthermore, morphological analysis of QN-PAES membranes (OH^−^ form) was carried out by SAXS, and the results are represented in Figure 8 and Table 5. As shown in Figure 8, the q_max_ of QN-PAES membranes (X10Y23, X10Y13, X19Y23, and X19Y13) were exhibited in the range of 0.91−0.98 nm^–1^, suggesting larger microphase-separated morphologies. The interdomain spacing (d) of membranes was calculated by Bragg’s equation based on the scattering vector [16,30]. The d values of QN-PAES membranes (X10Y23, X10Y13, X19Y23, and X19Y13) were 6.41, 6.54, 6.68, and 6.90 nm, respectively. The QN-PAES X19Y23 and X19Y13 membranes with longer hydrophilic length demonstrated comparatively clear scattering peaks because the QN-PAES X19Y23 and X19Y13 membranes have more quaternary ammonium groups and larger ion clusters than the QN-PAES X10Y23 and X10Y13 membranes. As expected, the morphology of QN-PAES membranes with controlled lengths of hydrophilic and hydrophobic blocks showed clear domain separation, proving that this strategy is an efficient method to facilitate phase separation. Therefore, these results of both AFM and SAXS reveal that increasing the lengths of hydrophilic blocks can be used to improve ion transportation channels.

### 3.7. Hydroxide Conductivity and Activation Energy

**Figure 9:**
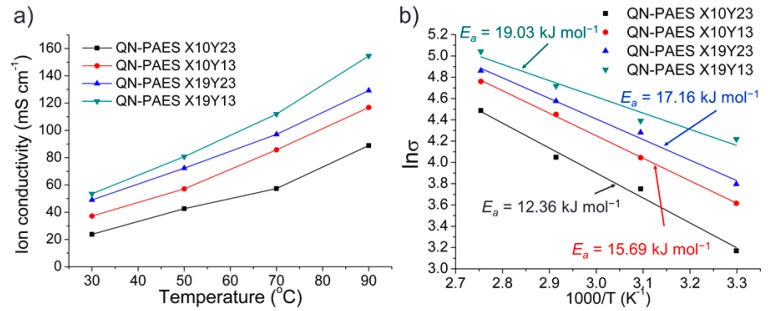
(**a**) The hydroxide conductivity of QN-PAES membranes and (**b**) Arrhenius plot of QN-PAES membranes at various temperatures under 100% RH.

**Figure 10:**
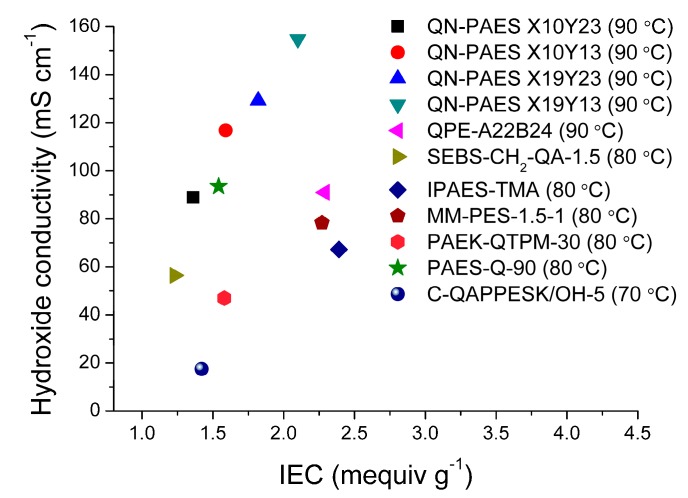
Hydroxide conductivity as a function of calculated IEC values’ comparison between recently reported AEMs.

The hydroxide conductivity of an AEM is one of the most important factors in determining the performance of AFCs. To investigate how the length of the hydrophilic oligomer and hydrophobic oligomer in the polymer affect hydroxide transfer, the hydroxide conductivity of QN-PAES membranes was measured at different temperatures under 100% RH. As shown in Figure 9a and Table 4, the hydroxide conductivity of membranes increased with increasing temperature due to the increasing activity and migration of ions through the expansion of ion channels [46,47,48]. As expected, the QN-PAES X19Y13 (154 mS cm^−1^ at 90 °C) containing longer hydrophilic oligomers and shorter hydrophobic oligomers showed the highest hydroxide conductivity compared to other QN-PAES membranes. Furthermore, to investigate the relationship between temperature (1000/T, Kelvin) and hydroxide conductivity (lnσ), the activation energy (*E*_a_, kJ mol^−1^) was calculated from the Arrhenius equation [49]. As shown in Figure 9b and Table 4, the fabricated QN-PAES membrane showed *E*_a_ values in the range from 12.4 to 19.0 kJ mol^−1^. The QN-PAES X19Y13 membrane showed lower activation energies than that of other QN-PAES membranes, which indicate increased hydrophilicity. A comprehensive comparison of IEC and hydroxide conductivity with reported AEMs is shown in Figure 10 and Table 6. Even though the IEC values (1.36–2.10 mequiv g^−1^) of fabricated QN-PAES membranes were similar to reported AEMs (1.23–2.39 mequiv g^−1^), the hydroxide conductivities of QN-PAES membranes were of higher value than the quaternary ammonium functionalized AEMs under 100% RH [18,50,51,52,53,54,55]. The improved electrochemical properties can be attributed to the excellent phase separation in the membrane caused by varying the hydrophilic and hydrophobic oligomer lengths in the polymer backbone.

### 3.8. Alkaline Stability

**Figure 11:**
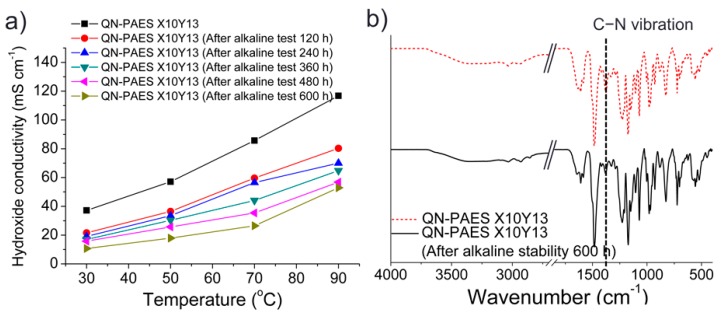
Alkaline stability of (**a**) QN-PAES X10Y13 membrane as a function of temperature and time, and (**b**) FT-IR spectra of QN-PAES X10Y13 membrane before and after alkaline stability test in a 2 M NaOH solution at 70 °C.

Apart from the high hydroxide conductivity and good thermal stability, alkaline stability is a major challenge for long-term use in alkaline fuel cells because AEMs based on quaternary ammonium groups have several drawbacks, such as degradation of the cationic groups under strong alkaline conditions. In this study, the alkaline stability of the QN-PAES membranes was evaluated by immersing the membranes in 2 M NaOH at 70 °C for 600 h. The hydroxide conductivity of QN-PAES X10Y23 and X10Y13 membranes (which maintained their appearance and flexibility) under these conditions was monitored, and the results are shown in Figure 11a and Figure S3, and Table S2. The hydroxide conductivity of membranes was assessed every 120 h interval up to 600 h. After the initial immersion in a 2 M NaOH solution at 70 °C for 120 h, the hydroxide conductivity of the QN-PAES X10Y13 membrane sharply decreased from 110.0 to 80.1 mS cm^−1^. Thereafter, the hydroxide conductivity of QN-PAES X10Y13 showed a slight decrease. Values were 69%, 60%, 55%, 49%, and 45% of the original hydroxide conductivity at intervals of 120 h, respectively. A comparison of FT-IR spectra before/after immersing membranes in a 2 M NaOH solution at 70 °C for 600 h is shown in Figure 11b. After the alkaline stability test, the absorption band at 1375 cm^−1^ and the stretching vibration of the C−N band tended to decrease, but there were no noticeable changes in the spectra. These results confirmed that the QN-PAES membranes are stable in the alkaline media, and are considered to be suitable for applications as AEMs.

### 3.9. Single Cell Performance

**Figure 12:**
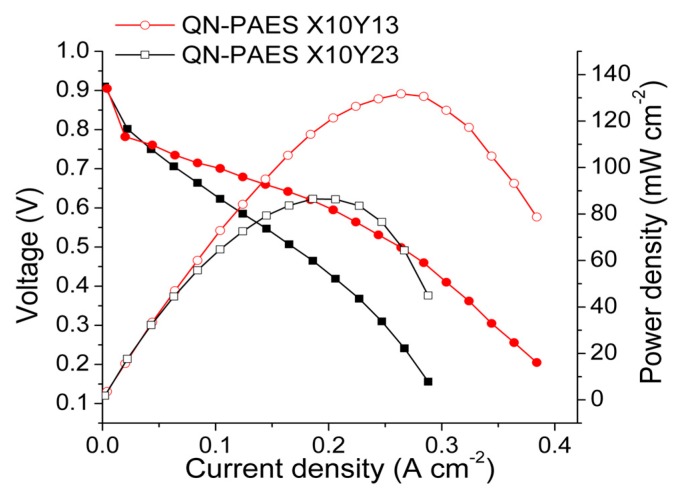
The polarization curves of QN-PAES X10Y23 and X10Y13 at 60 °C under fully humidified H_2_/O_2_ conditions.

The representative QN-PAES X10Y23 and X10Y13 having a good balance between hydroxide conductivity and dimensional stability were used to measure fuel cell performance. The fuel cell performance of QN-PAES X10Y23 and X10Y13 was investigated at 60 °C under 100% RH with H_2_/O_2_ fuel gas. The polarization and power density curves of the prepared MEA are shown in Figure 12. The OCV of the MEAs was 0.9 V, which means that the prepared membrane has low fuel permeability. The maximum power density of QN-PAES X10Y13 (131.7 mW cm^−2^) with shorter hydrophobic oligomer length is 1.5 times higher than that of X10Y23. In addition, the current density of QN-PAES X10Y13 was 1.6 times higher than that of X10Y23 at 0.6 V. The high fuel cell performance of QN-PAES X10Y13 is attributed to high hydroxide conductivity. These results indicate that increasing the hydrophilic oligomer length enhances the mobility of the ions, thereby improving the electrochemical properties. The maximum power density of the prepared MEA was high compared to the previous studies [56,57,58]. Therefore, considering the electrochemical performance and physical properties of the prepared QN-PAES X10Y13 membrane, it is considered to be an excellent candidate for an alkaline fuel cell.

## 4. Conclusions

A series of anion-conductive partially fluorinated multiblock poly(arylene ether sulfone)s (PAESs) containing quaternary ammonium groups were successfully synthesized with controlled lengths of the hydrophilic precursor and hydrophobic oligomer. Introduction of functional groups in CM-PAES and QN-PAES block copolymers was confirmed via ^1^H NMR and FT-IR. The fabricated QN-PAES membranes containing fluorine units were very flexible and transparent, and their thermal and dimensional stabilities were confirmed by TGA, water uptake, and swelling ratio. The AFM image reveals notable ion transport channels with increasing hydrophilic oligomer lengths in the main chains. In addition, morphological differences between the controlled hydrophilic and hydrophobic oligomer lengths were investigated by SAXS. The QN-PAES membranes showed excellent hydroxide conductivity. Moreover, after the alkaline stability test at 70 °C in a 2 M NaOH aqueous solution for 600 h, the chemical structure of QN-PAES membranes showed no noticeable change in FT-IR spectra, although there was a slight decrease in hydroxide conductivity. Overall, the QN-PAES membranes showed excellent electrochemical properties, and proved to be very useful as AEMs for AFCs.

## Figures and Tables

**Table 1:** Repeat units, molecular weights (*M*_n_ and *M*_w_) and PDI (*M*_w_/*M*_n_) of PAS and PAE oligomers.

Polymer	Repeat Unit		*M*_n_ (kDa)	*M*_w_ (kDa)	PDI (*M*_w_/*M*_n_)
	(a)	(b)			
PAS-X10	14	10	5.5	9.1	1.6
PAS-X19	25	19	10.7	17.0	1.6
PAE-Y13	−	13	7.9	18.5	2.3
PAE-Y23	−	23	14.5	25.7	1.7

(a) Calculated repeat unit by ^1^H NMR; (b) Calculated repeat unit by GPC.

**Table 2:** Molecular weights (*M*_n_ and *M*_w_) and PDI (*M*_w_/*M*_n_) of PAES block co-polymers.

Polymer	*M*_n_ (kDa)	*M*_w_ (kDa)	PDI (*M*_w_/*M*_n_)
PAES X10Y23	21.2	104.5	4.9
PAES X10Y13	20.3	124.2	6.1
PAES X19Y23	25.2	110.9	4.3
PAES X19Y13	19.8	87.5	4.4

**Table 3:** Thickness and mechanical behaviors of membranes.

Membrane	Thickness (µm)	Tensile Strength (MPa)	Young’s Modulus (GPa)	Elongation at Break (%)
QN-PAES X10Y23	28	77.6	3.4	1.8
QN-PAES X10Y13	27	67.4	2.3	2.7
QN-PAES X19Y23	25	62.3	1.0	6.9
QN-PAES X19Y13	26	60.5	0.9	10.9
QQBPES-2.4OH [33]	−	17.9	0.3	44.0
QPE-b [38]	52	48.1	0.7	54.0
Im-Am-PAEKS-3 [39]	−	34.5	1.4	3.6

**Table 4:** IEC, hydroxide conductivity, and activation energy (*E*_a_) of QN-PAES membranes.

Membrane	IEC (mequiv g^−1^)	Hydroxide Conductivity ^(a)^ (mS cm^−1^)				*E_a_* (kJ mol^−1^)
		30 °C	50 °C	70 °C	90 °C	
QN-PAES X10Y23	1.36	23.8	42.6	57.3	88.9	19.03
QN-PAES X10Y13	1.59	37.2	57.1	85.7	116.8	17.16
QN-PAES X19Y23	1.82	49.0	72.3	97.1	129.2	15.69
QN-PAES X19Y13	2.10	53.5	80.7	111.9	154.8	12.36

^(a)^ Under 100% RH.

**Table 5:** Scattering vector and Bragg spacing of the QN-PAES membranes calculated from SAXS data.

Membrane	Scattering Vector (q_max_, nm^–1^)	d-Spacing (nm)
QN-PAES X10Y23	0.98	6.41
QN-PAES X10Y13	0.96	6.54
QN-PAES X19Y23	0.94	6.68
QN-PAES X19Y13	0.91	6.90

**Table 6:** IEC and hydroxide conductivity values comparison with recently reported AEMs.

Membrane	IEC (mequiv g^−1^)	σ (mS cm^−1^)	Ref.
QN-PAES X10Y23	1.36	88.9 (90 °C)	this work
QN-PAES X10Y13	1.59	116.8 (90 °C)	this work
QN-PAES X19Y23	1.82	129.2 (90 °C)	this work
QN-PAES X19Y13	2.10	154.8 (90 °C)	this work
MM-PES-1.5-1	2.27	78.3 (80 °C)	[18]
QPE-A22B24	2.29	91.0 (90 °C)	[50]
SEBS-CH2-QA-1.5	1.23	56.4 (80 °C)	[51]
IPAES-TMA	2.39	67.2 (80 °C)	[52]
PAEK-QTPM-30	1.58	47.0 (80 °C)	[53]
PAES-Q-90	1.54	93.5 (80 °C)	[54]
C-QAPPESK/OH-5	1.42	17.5 (70 °C)	[55]

## Supplementary Materials

The following are available online at http://www.mdpi.com/2073-4360/10/12/1400/s1. Figure S1: ^1^H NMR spectra of (a) PAS-X10 and (b) PAS-X19; Figure S2: AFM height images of (a) QN-PAES X10Y23, (b) QN-PAES X10Y13, (c) QN-PAES X19Y23, and (d) QN-PAES X19Y13; Figure S3. Alkaline stability of QN-PAES X10Y23 membrane as a function of temperature and time in a 2 M NaOH solution at 70 ℃; Table S1: IEC, water uptake, swelling ratio (∆*x*, ∆*y*, and ∆*z*), and hydroxide conductivity of QN-PAES membranes; Table S2: Alkaline stability of QN-PAES membranes as a function of temperature and time in a 2 M NaOH solution at 70 ℃.

## References

[1] Liu X., Peng F.Y., Lou G.F. (2015). Liquid water transport characteristics of porous diffusion media in polymer electrolyte membrane fuel cells: A review. J. Power Sources.

[2] Dijoux E., Steiner N.Y., Benne M., Péra M.C., Pérez B.G. (2017). A review of fault tolerant control strategies applied to proton exchange membrane fuel cell systems. J. Power Sources.

[3] Ranjani M., Yoo D.J., Kumar G.G. (2018). Sulfonated Fe_3_O_4_@SiO_2_ nanorods incorporated sPVdF nanocomposite membranes for DMFC applications. J. Membr. Sci..

[4] Peighambardoust S.J., Rowshanzamir S., Amjadi M. (2010). Review of the proton exchange membranes for fuel cell applications. Int. J. Hydrog. Energy.

[5] Lee K.H., Chu J.Y., Kim A.R., Nahm K.S., Kim C.J., Yoo D.J. (2013). Densely sulfonated block copolymer composite membranes containing phosphotungstic acid for fuel cell membranes. J. Membr. Sci..

[6] Lee N.W., Duong D.T., Kim D.J. (2018). Cyclic ammonium grafted poly(arylene ether ketone) hydroxide ion exchange membranes for alkaline water electrolysis with high chemical stability and cell efficiency. Electrochim. Acta.

[7] Kraytsberg A., Ein-Eli Y. (2017). Review of advanced materials for proton exchange membrane fuel cells. Energy Fuels.

[8] Jannasch P., Weiber E.A. (2016). Configuring anion-exchange membranes for high conductivity and alkaline stability by using cationic polymers with tailored side chains. Macromol. Chem. Phys..

[9] Merle G., Wessling M., Nijmeijer K. (2011). Anion exchange membranes for alkaline fuel cells: A review. J. Membr. Sci..

[10] Varcoe J.R., Atanassov P., Dekel D.R., Herring A.M., Hickner M.A., Kohl P.A., Kucernak A.R., Mustain W.E., Nijmeijer K., Scott K. (2014). Anion-exchange membranes in electrochemical energy systems. Energy Environ. Sci..

[11] Dekel D.R. (2018). Review of cell performance in anion exchange membrane fuel cells. J. Power Sources.

[12] Hao J., Gao X., Jiang Y., Zhang H., Luo J., Shao Z., Yi B. (2018). Crosslinked high-performance anion exchange membranes based on poly(styrene-*b*-(ethylene-*co*-butylene)-*b*-styrene). J. Membr. Sci..

[13] Li N., Wang L., Hickner M. (2014). Cross-linked comb-shaped anion exchange membranes with high base stability. Chem. Commun..

[14] Tanaka M., Fukasawa K., Nishino E., Yamahuchi S., Yamada K., Tanaka H., Bae B.C., Miyatake M. (2011). Anion conductive block poly(arylene ether)s: Synthesis, properties, and application in alkaline fuel cells. J. Am. Chem. Soc..

[15] Rao A.H.N., Nam S.Y., Kim T.H. (2015). Comb-shaped alkyl imidazolium-functionalized poly(arylene ether sulfone)s as high performance anion-exchange membranes. J. Mater. Chem. A.

[16] Chu J.Y., Lee K.H., Kim A.R., Yoo D.J. (2019). Graphene-mediated organic-inorganic composites with improved hydroxide conductivity and outstanding alkaline stability for anion exchange membranes. Compos. Pt. B-Eng..

[17] Wang C., Xu C., Shen B., Zhao X., Li J. (2016). Stable poly(arylene ether sulfone)s anion exchange membranes containing imidazolium cations on pendant phenyl rings. Electrochim. Acta.

[18] Kwon S.H., Rao A.H.N., Kim T.H. (2018). Anion exchange membranes based on terminally crosslinked methyl morpholinium-functionalized poly(arylene ether sulfone). J. Power Sources.

[19] Li Y., Sniekers J., Malaquias J.C., Goethem C.V., Binnemans K., Fransaer J., Vankelecom I.F.J. (2018). Crosslinked anion exchange membranes prepared from poly(phenylene oxide) (PPO) for non-aqueous redox flow batteries. J. Power Sources.

[20] Li Q., Liu L., Miao Q., Jin B., Bai R. (2014). A novel poly(2,6-dimethyl-1,4-phenylene oxide) with trifunctional ammonium moieties for alkaline anion exchange membranes. Chem. Commun..

[21] Pan J., Han J., Zhu L., Hickner M.A. (2017). Cationic side-chain attachment to poly(phenylene oxide) backbones for chemically stable and conductive anion exchange membranes. Chem. Mat..

[22] Sajjad S.D., Hong Y., Liu F. (2013). Synthesis of guanidinium-based anion exchange membranes and their stability assessment. Polym. Adv. Technol..

[23] Lin X., Wu L., Liu Y., Ong A.L., Poynton S.D., Varcoe J.R., Xu T. (2017). Alkali resistant and conductive guanidinium-based anion-exchange membranes for alkaline polymer electrolyte fuel cells. J. Power Sources.

[24] Liu L., Li Q., Dai J., Wang H., Jin B., Rai R. (2014). A facile strategy for the synthesis of guanidinium-functionalized polymer as alkaline anion exchange membrane with improved alkaline stability. J. Membr. Sci..

[25] Xu Y., Ye N., Zhang D., Yang J., He R. (2017). Ionic crosslinking of imidazolium functionalized poly(aryl ether ketone) by sulfonated poly(ether ether ketone) for anion exchange membranes. J. Colloid Interface Sci..

[26] Yang J., Liu C., Hao Y., He X., He R. (2016). Preparation and investigation of various imidazolium-functionalized poly(2,6-dimethyl-1,4-phenylene oxide) anion exchange membranes. Electrochim. Acta.

[27] Gu F., Dong H., Li Y., Si Z., Yan F. (2014). Highly stable N3-substituted imidazolium-based alkaline anion exchange membranes: Experimental studies and theoretical calculations. Macromolecules.

[28] Hugar K.M., Kostalik H.A., Goates G.W. (2015). Imidazolium cations with exceptional alkaline stability: A systematic study of structure–stability relationships. J. Am. Chem. Soc..

[29] Long H., Pivovar B. (2014). Hydroxide degradation pathways for imidazolium cations: A DFT study. J. Phys. Chem. C.

[30] Zhang W., Liu Y., Jackson A.C., Savage A.M., Ertem S.P., Tsai T.H., Seifert S., Beyer F.L., Liberatore M.W., Herring A.M. (2016). Achieving continuous anion transport domains using block copolymers containing phosphonium cations. Macromolecules.

[31] Marino M.G., Kreuer K.D. (2015). Alkaline stability of quaternary ammonium cations for alkaline fuel cell membranes and ionic liquids. Chem. Sus. Chem..

[32] Tomé L.C., Guerreiro D.C., Teodoro R.M., Alves V.D., Marrucho I.M. (2018). Effect of polymer molecular weight on the physical properties and CO_2_/N_2_ separation of pyrrolidinium-based poly(ionic liquid) membranes. J. Membr. Sci..

[33] Dong X., Lv D., Zheng J., Xue B., Bi W., Li S., Zhang S. (2017). Pyrrolidinium-functionalized poly(arylene ether sulfone)s for anion exchange membranes: Using densely concentrated ionic groups and block design to improve membrane performance. J. Membr. Sci..

[34] Zha Y., Disabb-Miller M.L., Johanson Z.D., Hickner M.A., Tew G.N. (2012). Metal-cation-based anion exchange membranes. J. Am. Chem. Soc..

[35] Zhang X., Shi Q., Chen P., Zhou J., Li S., Xu H., Chen X., An Z. (2018). Block poly(arylene ether sulfone) copolymers tethering aromatic side-chain quaternary ammonium as anion exchange membranes. Polym. Chem..

[36] Kumar G.G., Kim A.R., Nahm K.S., Yoo D.J., Elizabeth E. (2010). High ion and lower molecular transportation of the poly vinylidene fluoride–hexafluoro propylene hybrid membranes for the high temperature and lower humidity direct methanol fuel cell applications. J. Power Sources.

[37] Yoo T.H., Aziz M.A., Oh K.J., Shanmugam S. (2017). Modified sulfonated poly(arylene ether) multiblock copolymers containing highly sulfonated blocks for polymer electrolyte membrane fuel cells. J. Membr. Sci..

[38] Tanaka M., Koike M., Miyatake K., Watanabe M. (2011). Synthesis and properties of anion conductive ionomers containing fluorenyl groups for alkaline fuel cell applications. Polym. Chem..

[39] Xu J., Liu B., Luo X., Li M., Zang H., Zhang H., Wang Z. (2017). Construction of ion transport channels by grafting flexible alkyl imidazolium chain into functional poly(arylene ether ketone sulfone) as anion exchange membranes. Int. J. Hydrog. Energy.

[40] Chu J.Y., Kim A.R., Nahm K.S., Lee H.K., Yoo D.J. (2013). Synthesis and characterization of partially fluorinated sulfonated poly(arylene biphenylsulfone ketone) block copolymers containing 6F-BPA and perfluorobiphenylene units. Int. J. Hydrog. Energy.

[41] Kim A.R., Vinothkannan M., Park C.J., Yoo D.J. (2018). Alleviating the mechanical and thermal degradations of highly sulfonated poly(ether ether ketone) blocks via copolymerization with hydrophobic unit for intermediate humidity fuel cells. Polymers.

[42] Dong X., Xue B., Qian H., Zheng J., Li S., Zhang S. (2017). Novel quaternary ammonium microblock poly(*p*-phenylene-*co*-aryl ether ketone)s as anion exchange membranes for alkaline fuel cells. J. Power Sources.

[43] Liu L., Ahlfield J., Tricker A., Chu D., Kohl P.A. (2016). Anion conducting multiblock copolymer membranes with partial fluorination and long head-group tethers. J. Mater. Chem. A.

[44] Lai A.N., Wang L.S., Lin C.X., Zhuo Y.Z., Zhang Q.G., Zhu A.M., Liu Q.L. (2015). Phenolphthalein-based poly(arylene ether sulfone nitrile)s multiblock copolymers as anion exchange membranes for alkaline fuel cells. ACS Appl. Mater. Interfaces.

[45] Xu J., Ma L., Han H., Ni H., Wang Z., Zhang H. (2014). Synthesis and properties of a novel sulfonated poly(arylene ether ketone sulfone) membrane with a high ꞵ-value for direct methanol fuel cell applications. Electrochim. Acta.

[46] Vinothkannan M., Kim A.R., Nahm K.S., Yoo D.J. (2016). Ternary hybrid (SPEEK/SPVdF-HFP/GO) based membrane electrolyte for the applications of fuel cells: Profile of improved mechanical strength, thermal stability and proton conductivity. RSC Adv..

[47] Lee K.H., Chu J.Y., Kim A.R., Yoo D.J. (2018). Facile fabrication and characterization of improved proton conducting sulfonated poly(arylene biphenylether sulfone) blocks containing fluorinated hydrophobic units for proton exchange membrane fuel cell applications. Polymers.

[48] Kim A.R., Vinothkannan M., Yoo D.J. (2017). Artificially designed, low humidifying organic-inorganic (SFBC-50/FSiO_2_) composite membrane for electrolyte applications of fuel cells. Compos. Part B Eng..

[49] Lee K.H., Chu J.Y., Kim A.R., Yoo D.J. (2018). Enhanced performance of a sulfonated poly(arylene ether ketone) block copolymer bearing pendant sulfonic acid groups for polymer electrolyte membrane fuel cells operating at 80% relative humidity. ACS Appl. Mater. Interfaces.

[50] Singh A.K., Pandey R.P., Shahi V.K. (2014). Fluorenyl phenolphthalein groups containing a multi-block copolymer membrane for alkaline fuel cells. RSC. Adv..

[51] Lin C.X., Wang X.Q., Hu E.N., Yang Q., Zhang Q.G., Zhu A.M., Liu Q.L. (2017). Quaternized triblock polymer anion exchange membranes with enhanced alkaline stability. J. Membr. Sci..

[52] Li X., Yu Y., Liu Q., Meng Y. (2013). Synthesis and characterization of anion exchange membranes based on poly(arylene ether sulfone)s containing various cations functioned tetraphenyl methane moieties. Int. J. Hydrog. Energy.

[53] Shen K., Zhang Z., Zhang H., Pang J., Jiang Z. (2015). Poly(arylene ether ketone) carrying hyperquaternized pendants: Preparation, stability and conductivity. J. Power Sources.

[54] Nie G., Li X., Tao J., Wu W., Liao S. (2015). Alkali resistant cross-linked poly(arylene ether sulfone)s membranes containing aromatic side-chain quaternary ammonium groups. J. Membr. Sci..

[55] Lu W., Shao Z.G., Zhang G., Li J., Zhao Y., Yi B. (2013). Preparation of anion exchange membranes by an efficient chloromethylation method and homogeneous quaternization/crosslinking strategy. Solid State Ion..

[56] Maurya S., Shin S.H., Kim M.K., Yun S.H., Moon S.H. (2013). Stability of composite anion exchange membranes with various functional groups and their performance for energy conversion. J. Membr. Sci..

[57] Kim E.Y., Lee S.J., Woo S.H., Park S.H., Yim S.D., Shin D.W., Bae B.C. (2017). Synthesis and characterization of anion exchange multi-block copolymer membranes with a fluorine moiety as alkaline membrane fuel cells. J. Power Sources.

[58] Zhang Z., Shen K., Lin L., Pang J. (2016). Anion exchange membranes based on tetra-quaternized poly(arylene ether ketone). J. Membr. Sci..

