# Supplementary material for: Improved Physicochemical Stability and High Ion Transportation of Poly(Arylene Ether Sulfone) Blocks Containing a Fluorinated Hydrophobic Part for Anion Exchange Membrane Applications
Source: Polymers (Basel). 2018 Dec 17;10(12):1400. doi: 10.3390/polym10121400 (PMC6401760; doi:10.3390/polym10121400)
Supplement: Supplementary file 1 [file polymers-10-01400-s001.pdf]

Supplementary Materials

Improved Physicochemical Stability and High Ion Transportation of Poly(Arylene Ether Sulfone) Blocks Containing a Fluorinated Hydrophobic Part for Anion Exchange Membrane Applications

Ji Young Chu [1], Kyu Ha Lee [1], Ae Rhan Kim [2,*] and Dong Jin Yoo [1,3,*]

[1] Department of Energy Storage/Conversion Engineering of Graduate School, Hydrogen and Fuel Cell Research Center, Chonbuk National University, Jeonju 54896, Republic of Korea; ebbuneg@hanmail.net (J.Y.C); carumiss@naver.com (K.H.L)

[2] R&D Center for CANUTECH, Business Incubation Center and Department of Bioenvironmental Chemistry, Chonbuk National University, Jeonju 54896, Republic of Korea

[3] Department of Life Science, Chonbuk National University, Jeonju 54896, Republic Korea

* Correspondence: canutech@hanmail.net (A.R.K); djyoo@jbnu.ac.kr (D.J.Y)

Received: 3 December 2018; Accepted: 15 December 2018; Published: 17 December 2018

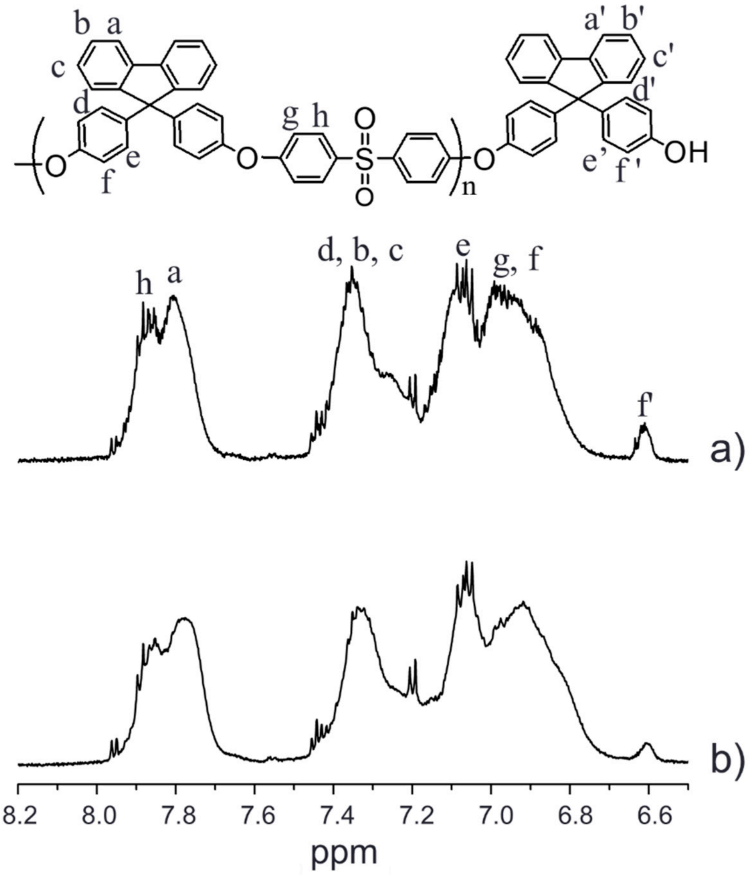

Figure S1. ^{1}H NMR spectra of a) PAS-X10 and b) PAS-X19.

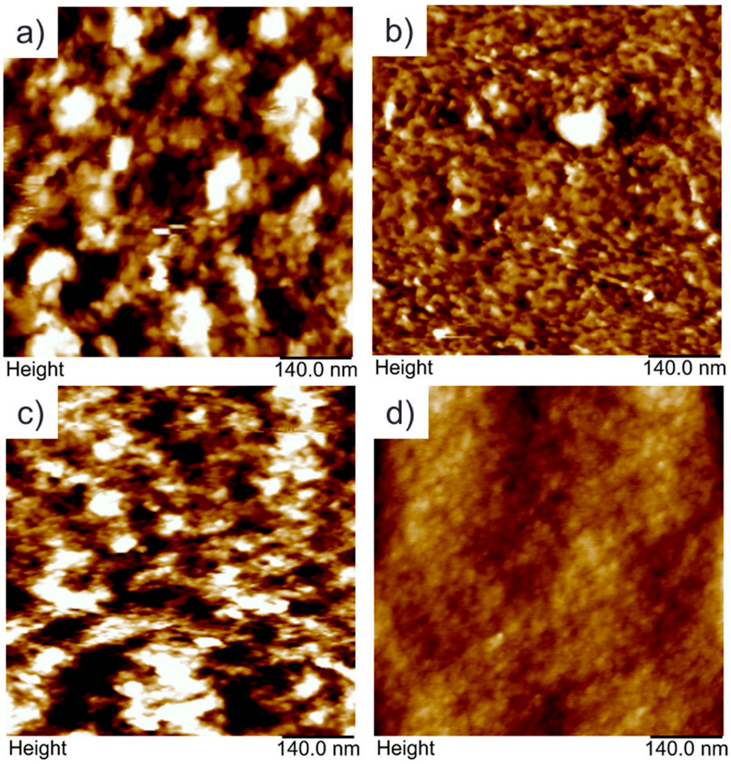

Figure S2. AFM height images of a) QN-PAES X10Y23, b) QN-PAES X10Y13, c) QN-PAES X19Y23, and d) QN-PAES X19Y13.

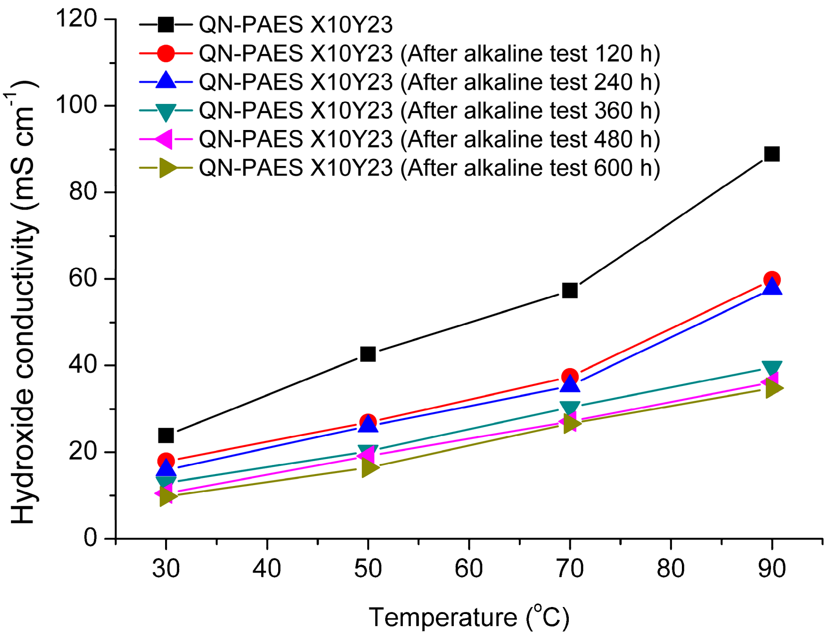

Figure S3. Alkaline stability of QN-PAES X10Y23 membrane as a function of temperature and time in a 2 M NaOH solution at 70 °C.

polymers

MDPI

Table 1. IEC, water uptake, swelling ratio (△*x*, △*y*, and △*z*), and hydroxide conductivity of QN-PAES membranes.

Membrane	IEC (mequiv g^{-1})	Water uptake (%)		Swelling ratio (△*x*, %)		Swelling ratio (△*y*, %)		Swelling ratio (△*z*, %)		Hydroxide conductivity at 90 °C (mS cm^{-1})
		30 °C	90 °C	30 °C	90 °C	30 °C	90 °C	30 °C	90 °C	
QN-PAES X10Y23	1.36	40.42	58.54	9.09	18.18	13.64	20.45	4.17	8.33	88.9
QN-PAES X10Y13	1.59	64.25	115.95	12.50	31.25	14.55	25.45	18.18	30.30	116.8
QN-PAES X19Y23	1.82	184.39	286.83	12.50	37.50	23.19	30.43	84.78	110.87	129.2
QN-PAES X19Y13	2.10	313.04	399.28	30.00	57.00	55.17	75.86	87.76	114.29	154.8

Table 2. Alkaline stability of QN-PAES membranes as a function of temperature and time in a 2 M NaOH solution at 70 °C.

Membrane	Hydroxide conductivity (mS cm^{-1}, 30 °C)		Hydroxide conductivity (mS cm^{-1}, 50 °C)		Hydroxide conductivity (mS cm^{-1}, 70 °C)		Hydroxide conductivity (mS cm^{-1}, 90 °C)	
	X10Y23	X10Y13	X10Y23	X10Y13	X10Y23	X10Y13	X10Y23	X10Y13
QN-PAES (initial)	23.8	37.2	42.6	57.1	57.3	85.7	88.9	116.8
QN-PAES (after 120 h)	17.9	21.6	26.9	36.5	37.4	59.6	59.8	80.3
QN-PAES (after 240 h)	15.9	19.1	26.0	33.5	35.3	56.5	57.8	70.0
QN-PAES (after 360 h)	12.9	17.0	20.2	30.3	30.4	44.1	39.6	64.8
QN-PAES (after 480 h)	10.5	15.8	19.1	25.7	27.1	35.4	36.2	56.8
QN-PAES (after 600 h)	9.8	10.7	16.4	17.9	26.6	26.5	34.8	52.9
